# Refractory Orbital Spindle Cell Carcinoma With Rapid Progression and Intracranial Extension

**DOI:** 10.1155/crop/9357403

**Published:** 2026-09-16

**Authors:** Elizabeth A. Bradley, Reza Nabie, Shadi Farabi Maleki

**Affiliations:** ^1^ Department of Ophthalmology, Mayo Clinic, Rochester, Minnesota, USA, mayoclinic.org; ^2^ Department of Ophthalmology, Nikookari Eye Hospital, Tabriz University of Medical Sciences, Tabriz, Iran, tbzmed.ac.ir

**Keywords:** carcinosarcoma, eye tumor, ocular oncology, orbit, spindle cell carcinoma

## Abstract

Spindle cell carcinoma, also known as carcinosarcoma or sarcomatoid carcinoma, is a rare and aggressive variant of squamous cell carcinoma known by biphasic malignant histology with epithelial and mesenchymal differentiation. We report the case of a 72‐year‐old male presenting with progressive unilateral proptosis, upper eyelid swelling, and restricted ocular motility. Imaging showed a well‐defined mass infiltrating multiple orbital structures, histopathologically supporting orbital spindle cell carcinoma after ruling out other mimics. After surgical debulking, followed by chemotherapy and radiotherapy, the tumor showed aggressive invasion with local infiltration, bone destruction, and intracranial extension, which ultimately led to the patient′s death. This case underscores detection and management challenges in the primary orbital spindle cell carcinoma. This tumor′s aggressiveness and resistance to traditional treatment approaches reveal the need for further investigation into its etiology and better management techniques.

## 1. Introduction

Spindle cell carcinoma, also referred to as carcinosarcoma, pseudosarcoma or sarcomatoid carcinoma, is an uncommon form of squamous cell carcinoma (SCC). It is defined by a dual malignant histological structure, involving both squamous and mesenchymal cell differentiation [1]. Spindle cell tumors are a type of connective tissue cancer marked by spindle‐shaped cells under the microscope. Although they frequently occur in the pleura, lungs, and other organs, they are rare in the head and neck [2]. Orbital involvement is uncommon and typically occurs via distant metastasis or local extension from adjacent structures. Because of the scarcity of cases, periocular spindle cell carcinoma′s behavior is still poorly understood [3]. Most studies report a higher prevalence in males and suggest that spindle cell carcinoma exhibits more aggressive behavior compared with conventional SCC [4]. However, the tumor′s behavior and potential for metastasis may vary depending on its site of origin; prognosis is generally poor [5]. Few cases of orbital spindle cell carcinoma have been documented, with most arising from distant metastases or regional spread from nearby structures like the paranasal sinuses. Reports of primary orbital spindle cell carcinoma are limited; however, a landmark primary presentation was documented by Jamshidian‐Tehrani et al. [6], who described a case of primary orbital spindle cell carcinoma presenting with limbal ischemia in a 61‐year‐old male. Other reported cases with orbital involvement did not originate primarily within the orbit, but rather presented as secondary local extensions from adjacent structures such as the maxillary sinus, nasal cavity, or paranasal sinuses [7, 8]. Clinically, spindle cell carcinomas of the orbit present with progressive unilateral proptosis, often painless but may cause vision impairment and eye movement restriction [9]. Some studies have shown that spindle cell carcinoma is more aggressive than regular SCC and is more likely to spread to distant parts of the body at the time of diagnosis [10]. Imaging techniques, including computed tomography (CT) and magnetic resonance imaging (MRI), help detect these well‐defined, vascularized tumors, whereas histopathology and immunohistochemistry (IHC) confirm the diagnosis [11]. Treatment primarily involves surgical excision, with close follow‐up, as radiotherapy or chemotherapy generally lacks efficacy in preventing recurrence [12]. This study presents a rare case of primary orbital spindle cell carcinoma and provides a brief review of the existing literature on this uncommon tumor.

## 2. Case Presentation

**Figure 1:**
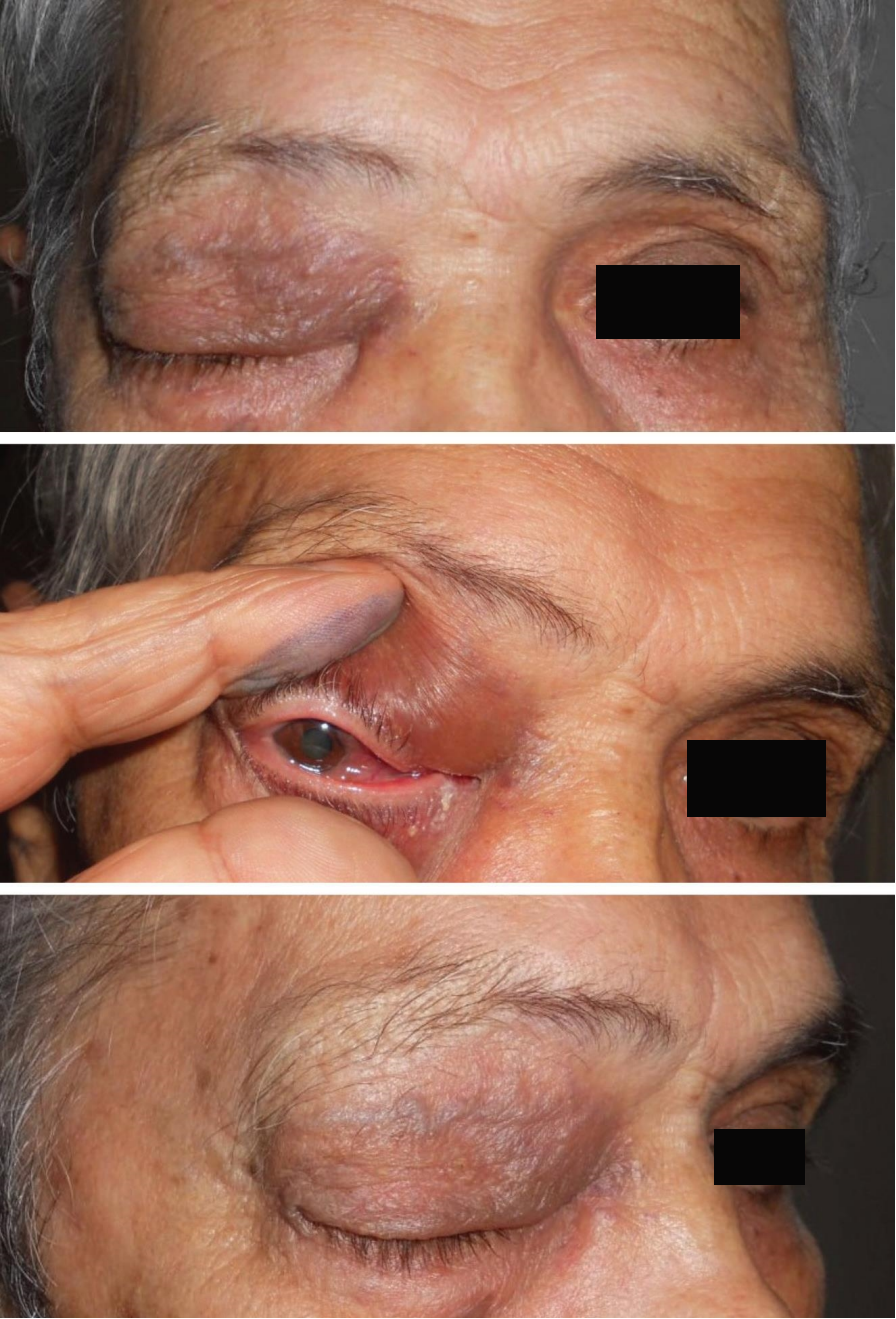
Preoperative external photographs of the patient reveal noticeable swelling and proptosis in the right upper eyelid.

A 72‐year‐old man presented with right eye periorbital swelling for 2 months. His best corrected visual acuity (BCVA) was 4/10 in the right eye and 6/10 in the left eye. Nuclear cataracts were present bilaterally. Otherwise, the examination of the left eye was unremarkable. Ophthalmic examination of the right eye revealed marked proptosis with severe upper eyelid edema and near‐complete mechanical ptosis, associated with conjunctival injection, chemosis, and prominent engorged conjunctival vessels. Extraocular motility was markedly restricted (Figure 1).

**Figure 2:**
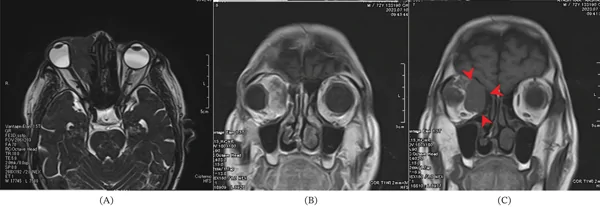
Preoperative MRI images of the patient in T1‐ and T2‐weighted sequences following gadolinium contrast administration. (A) Axial heavily T2‐weighted sequence MRI displaying a large, well‐defined, hypointense mass in the right orbit. (B) Coronal T1‐weighted noncontrast MRI showing the low‐signal intensity (hypointense) tumor in the superior‐medial right orbit. (C) Coronal T1‐weighted postcontrast (+IV gadolinium) MRI displaying heterogeneous enhancement of the mass. Red arrowheads delineate the multidirectional expansion of the mass, local tumor infiltration into the superior extraocular muscle complex, medial orbital wall, and lacrimal sac region.

MRI images revealed a very large well‐defined mass infiltrated the superior rectus, superior oblique and medial rectus muscles, the upper eyelid and the lacrimal sac. It was hypointense in both T1 and T2 and showed enhancement after gadolinium. The mass occupied a substantial portion of the orbit and was associated with marked proptosis and displacement of the globe (Figure 2).

**Figure 3:**
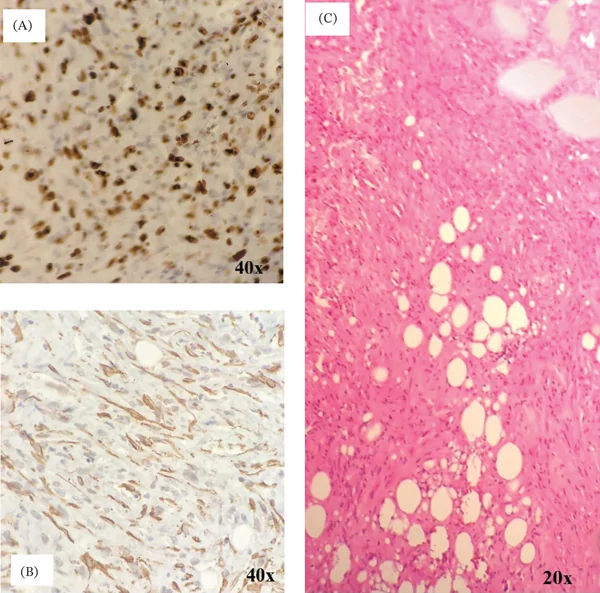
Histopathologic and immunohistochemical features of the tumor. (A) IHC showing diffuse nuclear positivity for Ki‐67 in approximately 50% of neoplastic cells, indicating a high proliferation index. (B) IHC showing S100 positivity in a subset of neoplastic spindle cells, with staining most prominent along elongated cytoplasmic processes. (C) Hematoxylin and eosin (H&E) stain demonstrating fascicles of spindle‐shaped neoplastic cells with moderate to severe nuclear pleomorphism.

The patient underwent orbitotomy via superior eyelid crease and Lynch incisions, and a dense whitish mass was debulked. Histopathological examination revealed a neoplastic proliferation of spindle cells with moderately pleomorphic spindle‐shaped nuclei, moderate amounts of cytoplasm, and frequent mitotic figures, approximately 10–12 per high‐power field (HPF). IHC stains were negative for desmin, SMA, CD 31, CD 34, CD 56, and HMB 45, whereas positive for scattered cells in S100 and 50% of cells in Ki67 (Figure 3). Although epithelial markers (PanCK and EMA) and SOX10 were unavailable due to institutional panel constraints, primary spindle cell carcinoma was established as the preferred diagnosis through immunophenotypic exclusion: Negative SMA and desmin excluded myogenic neoplasms, negative CD31/CD34/CD56 ruled out vascular lesions and solitary fibrous tumors, and negative HMB‐45 excluded melanoma.

**Figure 4:**
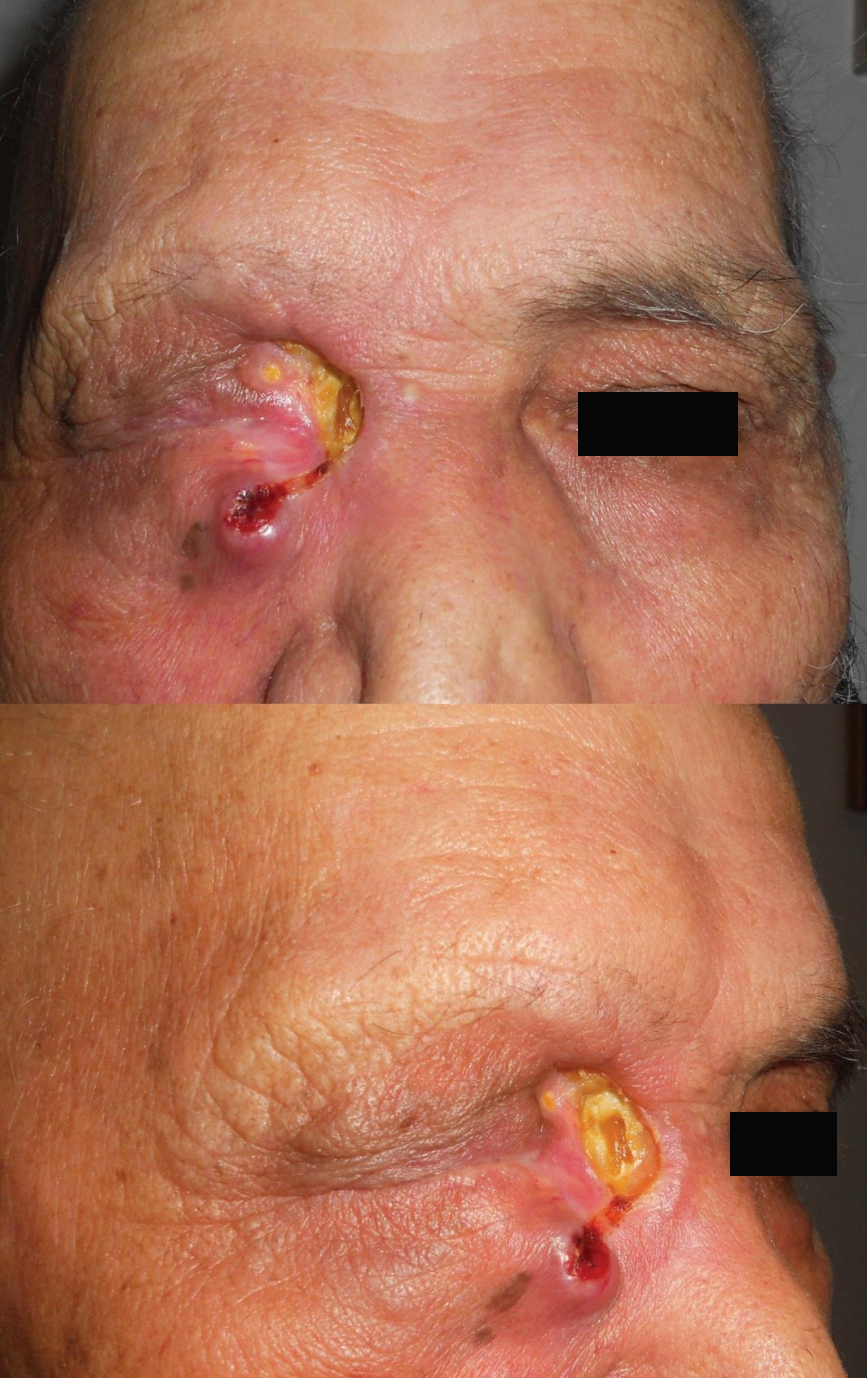
Postoperative external photographs taken 6 months after surgery show significant orbital infiltration with a vegetative ulcer at the lacrimal sac region.

The patient was referred to the oncology department for further evaluation. A comprehensive systemic workup, including positron emission tomography (PET) scanning, was subsequently performed and confirmed the primary orbital origin of the tumor, with no evidence of a systemic primary malignancy. Orbital exenteration was discussed with the patient; however, after counseling on the resulting facial deformity, he declined surgical exenteration. The chemotherapy and radiotherapy protocol were subsequently determined in consultation with radio‐oncologist. The radiotherapy dose was 55 Gy delivered in 28 fractions, and the chemotherapy protocol was dexamethasone 8 mg, Mesna 400 mg, granisetron 3 mg, doxorubicin 10 mg, ifosfamide 1000 mg, and pegfilgrastim 6 mg. The patient did not respond to chemotherapy, and by 1 month postoperatively, the incision site showed infiltration and ulceration. He suffered from severe dry eye with corneal haze and increased cataract density in the right eye that were attributed to radiotherapy. Six months after surgery, we observed increased infiltration of orbit with a vegetative ulcer at the lacrimal sac; corneal haze and cataract were increased and visual acuity decreased to counting fingers at 1 m. The patient was severely debilitated with restricted ocular motility and orbital pain (Figure 4).

**Figure 5:**
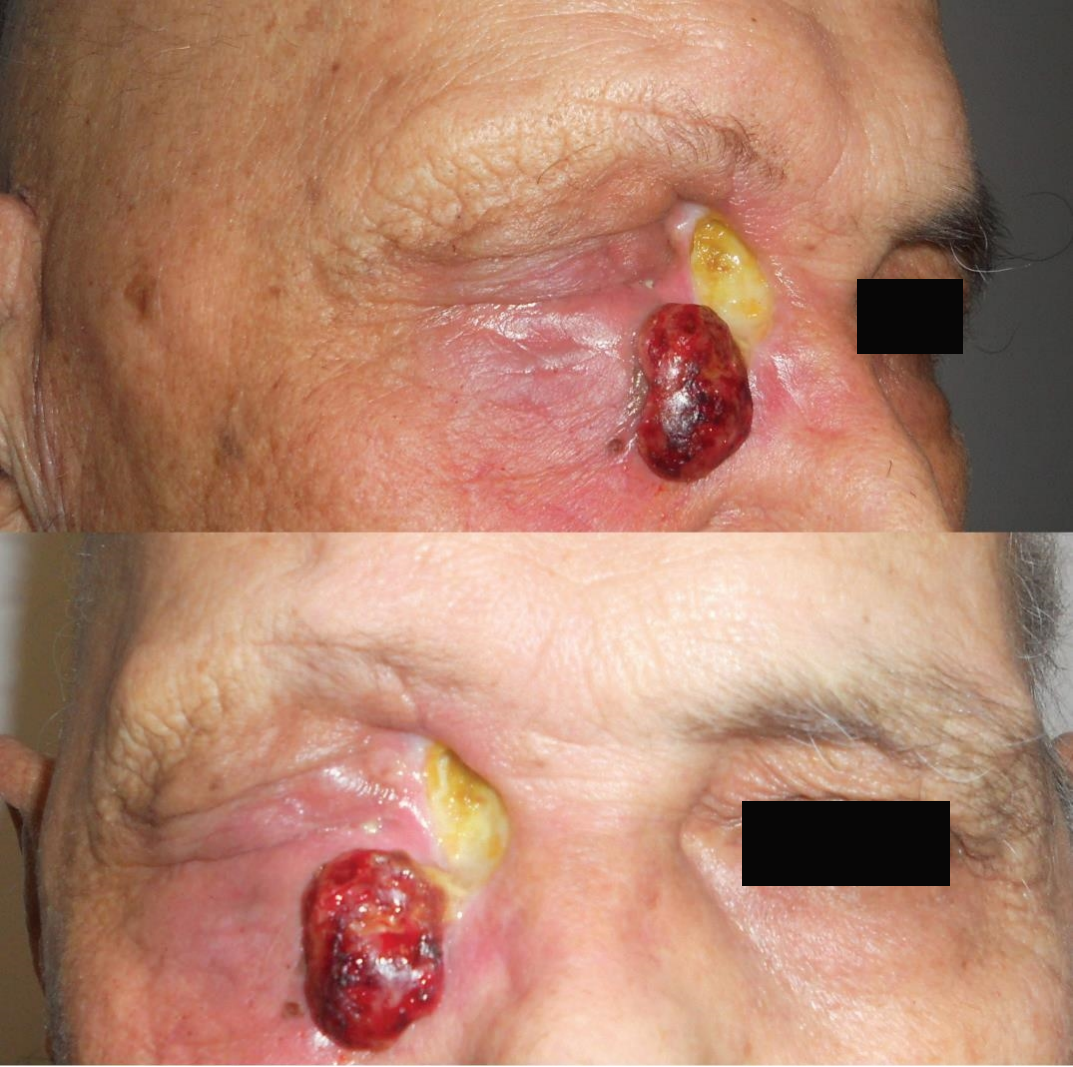
External photograph of the patient, 7 months postsurgery, showing a large infiltrative vegetative ulceration on the lower eyelid and cheek, along with skin necrosis in the lacrimal sac fossa.

Oncology and radiation therapy were again consulted, but no treatment was felt to offer tumor control. Palliative therapy was recommended. Seven months after surgery, the patient developed a large, infiltrative, and vegetative ulcerative lesion involving the lower eyelid and adjacent cheek, accompanied by extensive cutaneous necrosis in the region of the lacrimal sac fossa (Figure 5).

**Figure 6:**
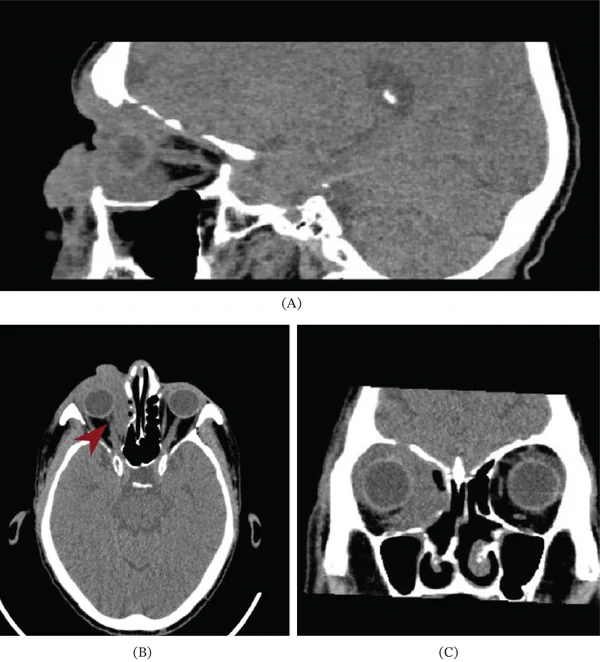
CT images of the patient, 7 months postsurgery. (A) Sagittal CT demonstrating extensive soft‐tissue tumor progression into the upper eyelid and superior orbital quadrants. (B) Axial CT showing a large anterior orbital mass with cutaneous/eyelid involvement. Red arrowhead highlights posterior intraorbital extension into the retrobulbar space. (C) Coronal CT displaying diffuse orbital infiltration across the superior, medial, and inferior quadrants with bone erosion and medial wall displacement into the ethmoid sinus.

Follow‐up CT demonstrated marked interval progression of the orbital mass, with extensive involvement of the superior, medial, and inferior orbital compartments and extension into the upper eyelid. The tumor was associated with destructive involvement of the superior and medial orbital walls, with displacement of the medial orbital wall toward the ethmoid sinus, indicating aggressive osseous invasion (Figure 6). The patient unfortunately was deceased 10 months after surgery due to intracranial tumor extension.

## 3. Discussion

Spindle cell carcinoma of the orbit is rarely reported, with known cases often linked to distant metastases or regional spread from paranasal sinuses. A 1988 study by Tabbara et al. [13] documented 10 patients with regional and distant metastasis, all of whom had delays in treatment. One patient developed metastases to the bone and lung. The aggressive progression in our case may be attributed to the delayed initiation of medical treatment and the tumor′s spindle cell variant.

Recently, Jamshidian‐Tehrani et al. [6] reported an orbital spindle cell tumor. It was determined that the patient had gastric adenocarcinoma with liver metastasis and was treated with chemotherapy. However, no specific relation was found between the adenocarcinoma and the orbital spindle cell tumor. It is believed that this biphasic tumor may result from sarcomatous transformation of conventional SCC [14]. Some primary orbital SCCs have occurred after retinal surgery, leading to the hypothesis that they may arise from the implantation of conjunctival epithelial cells and subsequent malignant degeneration [5]. Risk factors include smoking, alcohol, and previous radiotherapy [15]. It has been suggested that spindle cell carcinomas may arise from mesenchymal transformation of carcinoma cells or from a pluripotent stem cell differentiating into both epithelial and mesenchymal components. Since the orbit contains such stem cells, primary spindle cell carcinomas could result from their malignant degeneration [16].

The differential diagnosis of orbital spindle cell lesions is broad and includes spindle cell melanoma, monophasic synovial sarcoma, leiomyosarcoma, fibrosarcoma, solitary fibrous tumor, inflammatory myofibroblastic tumor, and dedifferentiated SCC [17]. In our case, considering the tumor′s spindle cell morphology, entities such as atypical neurofibroma and malignant peripheral nerve sheath tumors (MPNST) were considered. Atypical neurofibroma was excluded by the tumor′s highly aggressive clinical course and a high Ki‐67 proliferation index of 50%, which supports to a high‐grade malignancy [18]. Although the focal S100 positivity could raise suspicion for an MPNST, high‐grade MPNSTs characteristically lose diffuse S100 expression; however, the lack of an associated nerve origin, a negative CD34, and the absence of neurofibromatosis type 1 (NF1) history make MPNST less likely [19].

Most recently, Mason et al. [20] described a 68‐year‐old woman with a medial canthal lesion initially misdiagnosed as atypical fibroxanthoma, later confirmed as spindle cell carcinoma. The case demonstrated diagnostic challenges, surgical management, and the need for comprehensive evaluation and follow‐up. Similar to this case discussed in the Mason et al. study, our case also presented significant challenges. The patient was referred at an advanced stage of the disease, and the limited research on this topic raises questions about the best approach and treatment. Despite immediate surgical debulking and oncological therapies, the aggressive nature of this malignancy resulted in rapid tumor proliferation and spread, so our team faced several critical challenges: identifying the most accurate diagnostic strategy, selecting a biopsy method that would minimize the risk of tumor spread or metastasis, defining the appropriate imaging techniques, and deciding on a combination of treatments. These treatments included surgical debulking, chemotherapy, and radiotherapy, all of which required careful consideration to ensure the best outcome. By integrating this rare case to the documented data about spindle cell carcinoma cases, we are aimed at reducing clinical uncertainty, for establishing more streamlined diagnostic and therapeutic approaches, improving patient outcomes, and guiding clinicians toward effective evidence‐based decision‐making. This study discussed the importance of precise imaging, careful surgical planning, and a collaborative approach for the effective management of such cases.

Our primary limitation was a lack of specific antibody kits to test for epithelial markers like PanCK, SOX10, or EMA. However, our available panel successfully ruled out the main copycat tumors, including melanomas, vascular tumors, and muscle sarcomas. Therefore, primary orbital spindle cell carcinoma represents the most probable preferred diagnosis. Additional research with specific IHC staining is needed to better characterize this rare malignancy and optimize patient care.

## Author Contributions

E.A.B. managed and oversaw the study and critically reviewed the final manuscript; R.N. collected and managed the study data, performed data visualization, and handled manuscript submission; and S.F.M. drafted the initial manuscript and revised it to produce the final version.

## Funding

No funding was received for this manuscript.

## Ethics Statement

This case report does not meet the criteria for research involving human subjects that requires ethics committee approval, in accordance with local and national guidelines. Written informed consent was obtained from the patient for publication of the details of their medical case and any accompanying images. All patient data were fully anonymized, and the published images do not contain any identifiable features.

## Conflicts of Interest

The authors declare no conflicts of interest.

## Data Availability

The data that support the findings of this study are available from the corresponding authors upon reasonable request.
